# Differences in *Salmonella enterica* serovar Typhimurium strain invasiveness are associated with heterogeneity in SPI-1 gene expression

**DOI:** 10.1099/mic.0.048496-0

**Published:** 2011-07

**Authors:** Leann Clark, Charlotte A. Perrett, Layla Malt, Caryn Harward, Suzanne Humphrey, Katy A. Jepson, Isabel Martinez-Argudo, Laura J. Carney, Roberto M. La Ragione, Tom J. Humphrey, Mark A. Jepson

**Affiliations:** 1 School of Biochemistry, University of Bristol, University Walk, Bristol BS8 1TD, UK; 2 School of Cellular and Molecular Medicine, University of Bristol, University Walk, Bristol BS8 1TD, UK; 3 Department of Bacteriology, Veterinary Laboratories Agency, Weybridge, Woodham Lane, New Haw, Addlestone, Surrey KT15 3NB, UK; 4 Microbial Sciences Division, Faculty of Health and Medical Sciences, University of Surrey, Guildford, Surrey GU2 7XH, UK; 5 School of Veterinary Sciences, University of Bristol, Langford, Bristol BS40 5DU, UK

## Abstract

Most studies on *Salmonella enterica* serovar Typhimurium infection focus on strains ATCC SL1344 or NTCC 12023 (ATCC 14028). We have compared the abilities of these strains to induce membrane ruffles and invade epithelial cells. *S*. Typhimurium strain 12023 is less invasive and induces smaller membrane ruffles on MDCK cells compared with SL1344. Since the SPI-1 effector SopE is present in SL1344 and absent from 12023, and SL1344 *sopE* mutants have reduced invasiveness, we investigated whether 12023 is less invasive due to the absence of SopE. However, comparison of SopE^+^ and SopE^−^ *S*. Typhimurium strains, *sopE* deletion mutants and 12023 expressing a *sopE* plasmid revealed no consistent relationship between SopE status and relative invasiveness. Nevertheless, absence of SopE was closely correlated with reduced size of membrane ruffles. A P*prgH*–*gfp* reporter revealed that relatively few of the 12023 population (and that of the equivalent strain ATCC 14028) express SPI-1 compared to other *S*. Typhimurium strains. Expression of a P*hilA*–*gfp* reporter mirrored that of P*prgH*–*gfp* in 12023 and SL1344, implicating reduced signalling via the transcription factor HilA in the heterogeneous SPI-1 expression of these strains. The previously unrecognized strain heterogeneity in SPI-1 expression and invasiveness has important implications for studies of *Salmonella* infection.

## INTRODUCTION

The ability of *Salmonella enterica* to invade host cells, including normally non-phagocytic epithelial cells, is a critical virulence determinant. *Salmonella* invasion depends on *Salmonella* pathogenicity island 1 (SPI-1) which encodes a type III protein secretion system (TTSS-1), transcriptional regulators, effector proteins and chaperones. SPI-1-mediated invasion is considered to be dependent on actin rearrangements that promote cellular engulfment of the bacterium as a result of the action of several translocated effectors, including SopE, SopE2, SopB, SptP, SipA and SipC (McGhie *et al.*, 2009; Raffatellu *et al.*, 2005; Zhou & Galán, 2001). These proteins are translocated into the target cell via the TTSS-1 needle, a supramolecular complex composed of SPI-1-encoded proteins including PrgH, PrgK and InvG, each of which is essential for needle complex formation, effector translocation and SPI-1-stimulated invasion (Kubori *et al.*, 1998). Expression of SPI-1 genes is controlled by environmental signals via a complex regulatory network that converges on the transcriptional activator, HilA, and acts to control SPI-1 in an on/off manner (Ellermeier & Slauch, 2007; Temme *et al.*, 2008). Use of a transcriptional reporter, P*prgH–gfp^+^*, has confirmed that the *prgH* promoter, which drives transcription of the *prgHIJKorgABC* operon (Klein *et al.*, 2000), is only activated in subpopulations of *S. enterica* serovar Typhimurium (*S*. Typhimurium) cultures, resulting in marked population heterogeneity in invasiveness (Clark *et al.*, 2009; Hautefort *et al.*, 2003).

Biochemical activities have been ascribed to each of the effectors that play a major role in the invasion process. Apart from SipC, which is an essential component of the SPI-1 translocon, none of these effectors are necessary for invasion, due to some degree of redundancy in their activities (McGhie *et al.*, 2009). *Salmonella* outer protein E (SopE), which is only present in some virulent *Salmonella* strains, and its ubiquitous homologue SopE2 act as guanine nucleotide exchange factors (GEFs) for Rho GTPases (Hardt *et al.*, 1998; Temme *et al.*, 2008). SopB/SigD is an inositol polyphosphate phosphatase (Norris *et al.*, 1998) that, in addition to regulating membrane dynamics (Bujny *et al.*, 2008; Hernandez *et al.*, 2004; Terebiznik *et al.*, 2002), indirectly activates Rho GTPases (Patel & Galán, 2006), which promotes the actin rearrangements involved in *Salmonella* uptake. Each of the effectors that stimulate Rho GTPases (SopE, SopE2 and SopB) has additional roles in gut inflammation (Bruno *et al.*, 2009; Hardt *et al.*, 1998; Müller *et al.*, 2009). SptP has a GTPase-activating protein (GAP) activity that allows *Salmonella* to terminate membrane ruffling by switching off Rho GTPase activity (Fu & Galán, 1999). The cytoskeletal rearrangement stimulated by SopE, SopE2 and SopB is further regulated by the effectors SipA and SipC, two actin-binding proteins. SipC has been shown to directly nucleate actin polymerization and bundle filamentous actin (F-actin) (Hayward & Koronakis, 1999; McGhie *et al.*, 2001). SipA binds F-actin, promotes its formation, stabilization and bundling (McGhie *et al.*, 2004; Zhou *et al.*, 1999), and promotes *Salmonella* invasion in the initial stages of infection (Jepson *et al.*, 2001; Zhou *et al.*, 1999).

Almost all published studies on the molecular mechanisms of *Salmonella* invasion have focussed on just two strains of *S*. Typhimurium, ATCC SL1344 and NTCC 12023 (ATCC 14028), and almost all describe data obtained with just one of these strains. It is a common assumption that data obtained with one strain is representative of both the Typhimurium serovar and the *S. enterica* species as a whole. Nevertheless, some differences between SL1344 and 12023 have been noted that influence their interactions with host cells. For example, SL1344 carries a mutation in *hisG* which leads to the formation of filaments after invasion of some cell types that is not seen in 12023, which lacks this mutation (Henry *et al.*, 2005). The two most studied *S.* Typhimurium strains also differ in that the SPI-1 effector SopE is present in SL1344 and absent in 12023/14028. The presence of SopE has been associated with bovine epidemic strains, and a direct role for SopE in enteropathogenicity was demonstrated when phage-mediated transfer of *sopE* to 14028 was shown to increase fluid accumulation in ligated bovine gut loops (Zhang *et al.*, 2002). It is likely that SopE contributes to *Salmonella* enteropathogenicity via promotion of inflammatory responses (Zhang *et al.*, 2002). Strain 12023 has also been reported to be less invasive than SL1344 in HeLa cells (Unsworth *et al.*, 2004). The cause of the lower invasiveness of 12023 has not been directly examined, but reports that deletion of SopE from SL1344 reduces its invasiveness by 40–60 % (Hardt *et al.*, 1998; Mirold *et al.*, 2001; Stender *et al.*, 2000; Zhou *et al.*, 2001) suggest that the absence of SopE in 12023 might be a cause of reduced invasiveness. This possibility has not been addressed, and the relative importance of SopE in *Salmonella* invasion is itself unclear given the level of redundancy between effector protein functions and its absence from a large number of virulent serovars.

We tested the hypothesis that the lower invasiveness of strain 12023 is indicative of a major role for SopE in the relative levels of invasion of *Salmonella* strains. Comparison of SopE^+^ and SopE^−^ *S.* Typhimurium strains, *sopE* deletion mutants and 12023 expressing a *sopE* plasmid revealed no consistent relationship between invasion rates and SopE status. Nevertheless, absence of SopE was consistently associated with decreases in ruffle size and the speed at which *Salmonella* strains induce major rearrangement in plasma membrane architecture. Using a GFP reporter construct we found that only a small proportion of the 12023 population expresses the essential SPI-1 gene *prgH* under standard invasion-inducing conditions. A much higher proportion of the population of SL1344, and other *S.* Typhimurium strains tested, expressed P*prgH–gfp^+^*. Use of a similar GFP reporter for P*hilA* revealed that expression of this SPI-1-encoded transcriptional regulator is also much lower in strain 12023 than in SL1344. We suggest that decreased invasiveness of 12023 is due in large part to reduced expression of SPI-1 resulting from decreased activity of its transcriptional regulators.

## METHODS

### 

#### Bacterial strains and culture conditions.

Strains used in this study are listed in Table 1. Mutants of SL1344 and S1579/94 with in-frame deletions in *sopE* created by the Lambda Red system (Datsenko & Wanner, 2000) have been described previously (Perrett & Jepson, 2009). Complementation of SopE expression was achieved with the pACYC177-based plasmid pCAP03 (Perrett & Jepson, 2009). The P*prgH–gfp^+^* construct was transferred from SL1344 P*prgH–gfp^+^* (strain JH3010, kindly provided by J. Hinton and I. Hautefort, Institute of Food Research, Norwich, UK) to other *S*. Typhimurium strains (S1579/94, NCTC 12023, ATCC 14028, F98) by P22 transduction. Transductants were checked by PCR to confirm correct insertion.

**Table 1.** Bacterial strains and plasmids used in this study

Strain or plasmid	Description	Source or reference
***S*. Typhimurium**		
SL1344 (SopE^+^)	Virulent laboratory strain (*hisG* mutant of wild-type 4/74)	Hoiseth & Stocker (1981)
S1579/94 (SopE^+^)	Wild-type DT204 (horse isolate)	VLA strain collection
NCTC 12023 (SopE^−^)	Wild-type (NCTC deposition of ATCC 14028)	D. Holden
ATCC 14028 (SopE^−^)	Wild-type	C. Hueck
F98 (SopE^−^)	Wild-type	E. E. Galyov
SL1344 *sopE*^−^	SL1344 with in-frame deletion within *sopE*	C. M. A. Khan
S1579/94 *sopE^−^*	S1579/94 with in-frame deletion within *sopE*	Perrett & Jepson (2009)
SL1344 P*prgH–gfp*	SL1344 with transcriptional reporter fusion P*prgH–gfp^+^* (JH3010)	Hautefort *et al.* (2003)
S1579/94 P*prgH–gfp*	S1579/94 with transcriptional reporter fusion P*prgH–gfp^+^*	This study
12023 P*prgH–gfp*	NCTC 12023 with transcriptional reporter fusion P*prgH–gfp^+^*	This study
F98 P*prgH–gfp*	F98 with transcriptional reporter fusion *prgH–gfp^+^*	This study
14028 P*prgH–gfp*	ATCC 14028 with transcriptional reporter fusion P*prgH–gfp^+^*	This study
SL1344 P*hilA–gfp*	SL1344 with transcriptional reporter fusion P*hilA–gfp^+^*	This study
12023 P*hilA–gfp*	NCTC 12023 with transcriptional reporter fusion P*hilA–gfp^+^*	This study
SL1344 P*prgH–gfp* (Kan^R^)	SL1344 with chromosomal integration of P*prgH–gfp* from plasmid pSH04 (pZEP10 with Cm^R^ exchanged for Kan^R^)	This study
JH3016	SL1344 with transcriptional reporter fusion P*rpsM–gfp*	Hautefort *et al.* (2003)
**Plasmids**		
pTL61T	Promoterless *lacZ*; Amp^R^	Linn & St Pierre (1990)
pCAP03	pACYC177 carrying *sopE*; Amp^R^	Perrett & Jepson (2009)
pP*fliC–gfp*(LVA)	Transcription reporter for *fliC* linked to destabilized GFP	Ibarra *et al.* (2010)
pSH04	Transcriptional reporter for *prgH* linked to *gfp*^+^; Kan^R^ (pZEP10 derivative)	Humphrey *et al.* (2011)
pLM01	Transcriptional reporter for *hilA* linked to *gfp*^+^; Kan^R^ (pSH04 derivative)	This study
pSH08	Transcriptional reporter for *prgH* linked to *lacZ*	This study

To construct a chromosomal P*hilA–gfp* reporter construct the *hilA* promoter sequence was amplified from the *S.* Typhimurium SL1344 chromosome using primers HilA_F (5′-CATGCGCCCGGGCAGATGACACTATCTCCTTCC-3′) and HilA_R (5′-GATCTCTAGAGTATAATAGTGTATTCTCTT-3′) adapted from Boddicker & Jones (2004). The promoter was amplified via PCR using Roche's Expand High Fidelity enzyme, amplifying a section of the chromosome −496 to +350 of *hilA*. The PCR product was digested with *Xba*I and *Xma*I, purified and ligated into the *Xba*I/*Xma*I-digested pSH04 vector (Humphrey *et al.*, 2011) containing a *gfp^+^* reporter gene and kanamycin selection. The P*hilA–gfp^+^–kan*^R^ construct was amplified from the ligated plasmid using primers T1_F1 (5′-GCAGGTCACATTTAACGCGGTTGCACAAGTTGCAACATGGCCTGGGGTAATGACTCTCTAGC-3′) from Hautefort *et al.* (2003) and KanR_R3 (5′-GACCCGGATAGTAATTTTGCCCGGCCAGATGATAAATCGCGACACGCTCAGAAGAACTCGTCAAGAAGG-3′). These primers had flanking regions with homology to the *putPA Salmonella* locus, previously shown to have no effect on invasion in *Salmonella*. This construct was integrated into the SL1344 chromosome using the Lambda Red technique as originally described by Datsenko & Wanner (2000) and modified by Hautefort *et al.* (2003). Recombinants were selected on LB agar with kanamycin, and verified by PCR. Finally, the construct was transferred into SL1344 and 12023 via P22 transduction.

Prior to analysis of invasiveness and other assays, strains were grown overnight at 37 °C as a static culture in LB (Miller) broth supplemented with antibiotics as appropriate (carbenicillin 100 µg ml^−1^, chloramphenicol 15 µg ml^−1^ and kanamycin 100 µg ml^−1^); cultures were then diluted 1 : 100 into LB and grown for appropriate times at 37 °C in an orbital shaker (150 r.p.m.), 3.5 h (late-exponential phase) being the standard growth period used to optimize invasion and SPI-1 expression.

#### Infection of cultured cells.

MDCK strain I cells were grown in Eagle's Minimum Essential Medium (EMEM) supplemented with 10 % (v/v) fetal calf serum, 1 % (v/v) non-essential amino acids, 1 % GlutaMAX and 100 µg kanamycin ml^−1^ at 37 °C in a humidified atmosphere of 5 % CO_2_. When confluent, cells were passaged and seeded onto 13 mm coverslips (2.5×10^4^ cells per coverslip). Three days after seeding, the medium was replaced with a modified Krebs' buffer (mM: 137 NaCl, 5.4 KCl, 1 MgSO_4_, 0.3 KH_2_PO_4_, 0.3 NaH_2_PO_4_, 2.4 CaCl_2_, 10 glucose and 10 Tris, adjusted to pH 7.4 at 37 °C with HCl). After equilibration in this medium at 37 °C in air, *S.* Typhimurium was added at an m.o.i. of 20–50. To compare the effect of centrifugation on infection, cells grown as above were infected with *Salmonella* and subjected to mild centrifugation (650 ***g***, 5 min, room temperature) in tissue-culture plates. After centrifugation, cells were placed in a 37 °C incubator for 15 min.

#### Quantification of invasion.

After infection of MDCK cells with *S*. Typhimurium strains for 15 min, invasion was quantified using differential immunofluorescent staining as described by Perrett & Jepson (2007). Quantification of MDCK cells (DAPI-labelled), and adherent (FITC-labelled) and total (TRITC-labelled) bacteria, was achieved by blind scoring of at least 10 random fields as previously described (Perrett & Jepson, 2009).

#### Analysis of membrane ruffling by cytochemical staining of F-actin.

After infection of cells for 15 min, actin rearrangement and the location of *Salmonella* were determined with goat anti-*Salmonella* CSA-1 antibody and FITC-conjugated rabbit anti-goat immunoglobulin alongside TRITC-phalloidin, as described previously (Perrett & Jepson, 2009). Membrane ruffles induced by *Salmonella* were examined and counted by blind scoring of at least 10 random fields as previously described (Perrett & Jepson, 2009). Images of TRITC-phalloidin (F-actin) and FITC immunostaining (*Salmonella*) were obtained by confocal laser-scanning microscopy (Perrett & Jepson, 2009). Comparison of ruffle size was achieved by measuring the longest diameter of all membrane ruffles in at least two randomly selected fields of view from two replicate experiments.

#### Phase-contrast time-lapse microscopy.

To study the dynamics of membrane ruffling, MDCK cells grown on 22 mm coverslips were infected with *Salmonella* strains during acquisition of time-lapse sequences (10 s intervals) of phase-contrast and fluorescence images using a wide-field imaging system as described previously (Perrett & Jepson, 2009). The image sequences were acquired and analysed using Openlab 4 software (Improvision) and the timing of all adherence and ruffling events within random fields of view was recorded to determine the time taken to induce membrane ruffles.

#### Analysis of GFP reporter strains.

Overnight cultures of strains containing the chromosomal P*prgH–gfp^+^* construct were diluted 1 : 100 in LB and grown for up to 6 h at 37 °C in an orbital shaker. At fixed time points, samples were examined with a haemocytometer and a Leica DM LB2 microscope using a ×63 phase-contrast objective, and the proportion of green fluorescent bacteria was calculated (Clark *et al.*, 2009). Phase-contrast and fluorescence images were acquired on a wide-field imaging system as previously described (Clark *et al.*, 2009). The P*hilA* reporter strains were grown as above and analysed by flow cytometry after fixation by centrifuging 0.5 ml of culture at 3293 ***g*** for 2 min, resuspending in equal volumes of 2 % paraformaldehyde (w/v in PBS) and storage at 4 °C until required. Fixed samples were diluted 1 : 50 in FACSflow buffer (BD Biosciences), and flow cytometric analysis was conducted with an LSR II flow cytometer (BD Biosciences). Data acquisition (approx 100 000 events per sample) and analysis were performed using Diva 6.1.2 software, with native GFP^−^ *S*. Typhimurium SL1344 and constitutively GFP^+^ SL1344 (JH3016; P*rpsM–gfp*) being used as gating controls.

#### Construction and application of a transcriptional P*prgH*–*lacZ* reporter.

In order to investigate the activity of the *prgH* promoter in different *S*. Typhimurium strains, a transcriptional P*prgH–lacZ* reporter fusion was created. Briefly, using oligonucleotides PprgH_F1 (5′-ACGTCCCGGGGATGACTATTACTTACAAAGG-3′) and PprgH_R1 (5′-CGATTCTAGACGAACTATGTATGGCCCTGG-3′) (Hautefort *et al.*, 2003), the *prgH* promoter region was amplified from the *S.* Typhimurium SL1344 chromosome by PCR under standard conditions. The resulting PCR product was digested with *Xma*I and *Xba*I, and ligated into the backbone of *Xma*I/*Xba*I-digested pTL61T vector (Linn & St Pierre, 1990). Ligation products were transformed into *Escherichia coli* DH5α (Invitrogen) according to the manufacturer's instructions, with selection of putative transformants on LB agar supplemented with carbenicillin at 100 µg ml^−1^, X-Gal at 40 µg ml^−1^ and 0.2 mM IPTG. Putative transformants were selected and their plasmids verified by sequencing, generating plasmid pSH08. Finally, pSH08 was transformed into *S*. Typhimurium SL1344 and 12023 by electroporation.

To compare P*prgH–lacZ* activity, SL1344 and 12023 carrying pSH08 were grown overnight in standard Miller LB supplemented with 100 µg carbenicillin ml^−1^. Cultures were subsequently subcultured 1 : 100 (v/v) into LB, grown for 3.5 h at 37 °C, 150 r.p.m., and their OD_600_ measured. To determine the β-galactosidase activity, 50 µl of culture (*V*) was added to 950 µl lysis buffer [100 ml Buffer Z, 50 µl 10 % SDS and 270 µl β-mercaptoethanol (Sigma-Aldrich)], and then 20 µl choloroform was added to lyse the cells. The mixture was vortexed for 10 s, and incubated statically at 30 °C for 10 min. The reaction was initiated by addition of 200 µl ONPG (Sigma-Aldrich) substrate (4 mg ml^−1^ in Buffer Z), and all samples were subsequently incubated at 30 °C in the dark. The time taken (*T*) for the solution to develop a yellow colour was recorded, and the reaction terminated by addition of 0.5 ml 1 M Na_2_CO_3_. Samples were centrifuged at 11 350 ***g*** for 5 min to remove debris, and 1 ml supernatant transferred to a cuvette for measurement of absorbance at 420 and 550 nm. Activity of β-galactosidase was calculated in Miller units (Miller, 1972).

#### Statistical analysis.

Unless stated otherwise, all data are expressed as means±sem, and statistical significance was assessed using an unpaired, two-way Student's *t*-test with significance set at *P*≤0.05.

## RESULTS

### Variation of *S*. Typhimurium strains in epithelial cell invasion and membrane ruffle formation

**Fig. 1.**
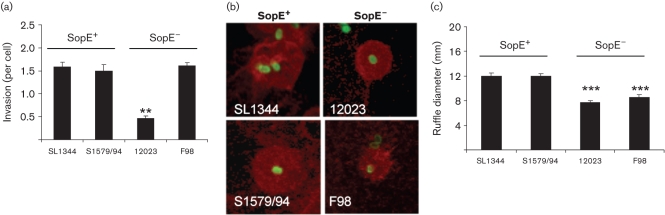
Comparison of invasion and membrane ruffling associated with *S*. Typhimurium strains. *Salmonella* strains naturally SopE^+^ (SL1344 and S1579/94) or SopE^−^ (F98, 12023) were grown at 37 °C, 150 r.p.m., for 3.5 h and used to infect MDCK cells grown on coverslips at an m.o.i. of ~50 for 15 min. (a) Differential immunolabelling was used to determine invasion; data are from four independent experiments, expressed as means±sem. (b) Invasion by strain 12023 was significantly lower than that by the three alternative wild-type strains. Fluorescent labelling of membrane ruffles localized by F-actin labelling with TRITC-phalloidin (red) and *Salmonella* by immunolabelling (green) reveals that both naturally SopE^−^ strains induce smaller membrane ruffles than those triggered by SopE^+^ strains. Representative confocal images of each strain; field of view 16 µm×16 µm. (c) Analysis of membrane ruffle diameter from confocal images reveals that significantly smaller ruffles are induced by the two SopE^−^ strains. Measurements of all membrane ruffle diameters in randomly selected fields of view expressed as means±sem (*n* = 34–84). Significant differences from both SopE^+^ wild-type strains indicated by asterisks: **, *P*<0.01; ***, *P*<0.00001.

Infection of MDCK cells revealed that *S.* Typhimurium strain 12023 was significantly less invasive, and induced smaller membrane ruffles, than strain SL1344 (Fig. 1). Since others have shown that deletion of *sopE* from SL1344 impairs invasion (Hardt *et al.*, 1998; Mirold *et al.*, 2001; Stender *et al.*, 2000; Zhou *et al.*, 2001), we tested the hypothesis that the low invasion associated with 12023 infection is due to its lacking SopE. Comparison with two other Typhimurium strains whose SopE status had previously been confirmed as either *sopE*^+^ (S1579/94) or *sopE*^−^ (F98) (Perrett & Jepson, 2009) revealed that 12023 is significantly less invasive than both these (Fig. 1a).

Microscopical analysis of membrane ruffles identified by TRITC-phalloidin labelling of F-actin revealed that 12023 induced fewer ruffles than the three other strains, consistent with its lower invasiveness (data not shown). Analysis of the size of membrane ruffles revealed that both SopE^−^ wild-type strains (12023 and F98) induced ruffles that were on average significantly smaller than those induced by the two SopE^+^ strains (SL1344 and S1579/94) (Fig. 1b, c).

Thus, while there was no consistent relationship between the SopE status of wild-type strains and their invasiveness, there may be a direct relationship between the presence of SopE and the size of membrane ruffles.

### Deletion of SopE has a consistent impact on membrane ruffling but not on invasion

**Fig. 2.**
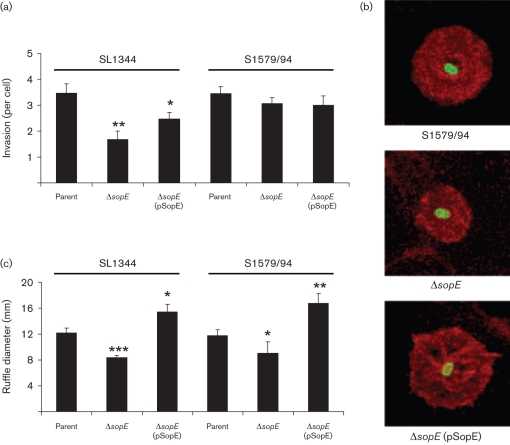
Comparison of invasion and membrane ruffling induced by wild-type and *sopE* mutant *S*. Typhimurium strains. Naturally SopE^+^ *Salmonella* strains (SL1344 and S1579/94), *sopE* mutants and plasmid-complemented mutants were grown at 37 °C, 150 r.p.m., for 3.5 h and used to infect MDCK cells grown on coverslips at an m.o.i. of ~50 for 15 min. (a) Invasion was assessed by differential immunolabelling in six (SL1344) or four (S1579/94) independent experiments and data are expressed as means±sem. Note that invasion is significantly reduced by *sopE* deletion from SL1344 but not from S1579/94. (b) Fluorescent labelling of membrane ruffles with TRITC-phalloidin (red) and *Salmonella* by immunolabelling (green) reveals that deletion of *sopE* from S1579/94 is associated with induction of membrane ruffles that are generally smaller than those induced by parent and SopE^−^ complemented strains. Confocal images; field of view 16 µm×16 µm. (c) Measurement of all membrane ruffles in randomly selected fields of MDCK cells infected with parent SopE^+^ strains (SL1344 and S1579/94), *sopE* mutants or SopE^−^ complemented strains reveals a significant decrease in ruffle diameter associated with *sopE* deletion that is complemented by expression of SopE *in trans*. Data are expressed as means±sem (*n* = 30–59 for SL1344 and *n* = 16–29 for S1579/94). Significant differences from parent strains are indicated by asterisks: *, *P*<0.05; **, *P*<0.01; ***, *P*<0.00001.

We further assessed the impact of SopE on *Salmonella* interactions with epithelial cells by comparing naturally SopE^+^ strains, *sopE* mutants, and complemented versions thereof. These comparisons confirmed a lack of association between SopE status and invasion. While deletion of *sopE* rendered SL1344 52 % less invasive in MDCK cells (confirming previous observations in other cell lines), there was no effect on invasion of S1579/94 when *sopE* was deleted (Fig. 2a). These findings reveal a previously unrecognized heterogeneity in the impact of SopE on invasiveness of *S*. Typhimurium strains.

Despite the lack of correlation between SopE and invasion detected in our assays, there was a consistent effect of *sopE* deletion on the size of membrane ruffles induced by both SL1344 and S1579/94. Confocal images of typical membrane ruffles induced by wild-type, *sopE* mutant and complemented versions of strain S1579/94 are shown in Fig. 2(b). Analysis of the diameter of membrane ruffles confirmed a significant decrease in ruffle size following *sopE* deletion, which is fully reversed, and indeed surpasses that of the wild-type, when SopE is complemented *in trans* with plasmid pCAP03 (Fig. 2c). The reduced invasiveness of the SopE^−^ strain 12023 (Fig. 1b) prompted us to investigate the effect of plasmid expression of SopE in this strain. The SopE plasmid pCAP03 significantly increased ruffle size induced by 12023 from 5.3±0.5 µm to 13.1±0.6 µm (*P*<0.0001). Invasion of 12023 was also increased by expression of SopE but remained significantly lower (*P*<0.0001) than that of SL1344 (data not shown). Live cell imaging confirmed the size difference between ruffles induced by SopE^+^ and SopE^−^ strains and also revealed a direct relationship between the presence of SopE and the speed with which membrane ruffles are induced after *S*. Typhimurium strains make contact with the epithelial cell plasma membrane (Table 2). Taken together, these data support the view that SopE contributes to membrane ruffling while decreased invasion of 12023 involves additional contributory factors.

**Table 2.** Comparison of membrane ruffling in SopE^−^ and SopE^+^ *Salmonella* strains by live cell imaging Analysis of membrane ruffling by live cell imaging reveals differences between SopE^+^ strains (larger ruffles triggered on average more rapidly) and SopE^−^ strains (ruffles tend to be smaller and induced more slowly). Ruffle size data are expressed as mean peak diameter±sem; *n* = 30; significant differences from both SopE^+^ strains were assessed by one-way ANOVA and Tukey's post-test (*, *P*<0.05). Ruffle induction times were not normally distributed and are shown as median values with ranges in parentheses. Significant differences from both SopE^+^ strains were assessed by Kruskal–Wallis test and Dunn's multiple comparison test (†, *P*<0.05).

	SopE^+^		SopE^−^		
	SL1344	S1579/94	12023	F98	SL1344 *sopE*-
Peak ruffle diameter (µm)	8.38±0.38	8.55±0.70	5.99±0.28*	6.17±0.37*	5.97±0.09*
Ruffle induction time (s)	40 (10–290)	40 (10–310)	70 (10–320)†	60 (20–270)†	65 (10–330)†

### Low invasiveness of *S*. Typhimurium strain 12023 is correlated with decreased *prgH* promoter activity

Having demonstrated that there is no consistent relationship between SopE status and invasiveness of *S.* Typhimurium strains, we tested the alternative hypothesis that the lower invasiveness of 12023 might be related to a difference in regulation of SPI-1 expression. To investigate this possibility, we used a previously described P*prgH–gfp* chromosomal insertion, a transcriptional reporter for the SPI-1 gene *prgH* which encodes a structural component located within the inner ring of the needle complex (Schraidt *et al.*, 2010). Previous work has revealed that only a subpopulation of SL1344 P*prgH–gfp* expresses GFP under all conditions tested thus far, the proportion reaching 53–70 % during late-exponential-phase growth, conditions we and others use to optimize invasion (Clark *et al.*, 2009; Hautefort *et al.*, 2003; Perrett *et al.*, 2009). To confirm that the P*prgH–gfp* construct is a reliable reporter of the invasive phenotype, we examined the GFP expression status of individual SL1344 P*prgH–gfp* infecting MDCK epithelial cells by live cell imaging and by fluorescent labelling of fixed cells. These studies revealed that only GFP-expressing bacteria induced ruffles, although some non-expressing individuals were occasionally taken up into cells via the membrane ruffles triggered by GFP-expressing bacteria (data not shown). This latter observation is consistent with previous reports that ruffles induced by invasive *Salmonella* can drive internalization of non-invasive bacteria (Francis *et al.*, 1993; Jepson *et al.*, 2001). Quantification of bacteria by immunofluorescent labelling revealed that 98.9±1.4 % of cell-associated SL1344 were P*prgH–gfp* positive. The latter data are consistent with recent reports that the SPI-1 TTSS promotes adherence to epithelial cells (Lara-Tejero & Galán, 2009; Misselwitz *et al.*, 2011). Together, these data demonstrate a positive correlation between P*prgH–gfp* expression and the infective phenotype.

**Fig. 3.**
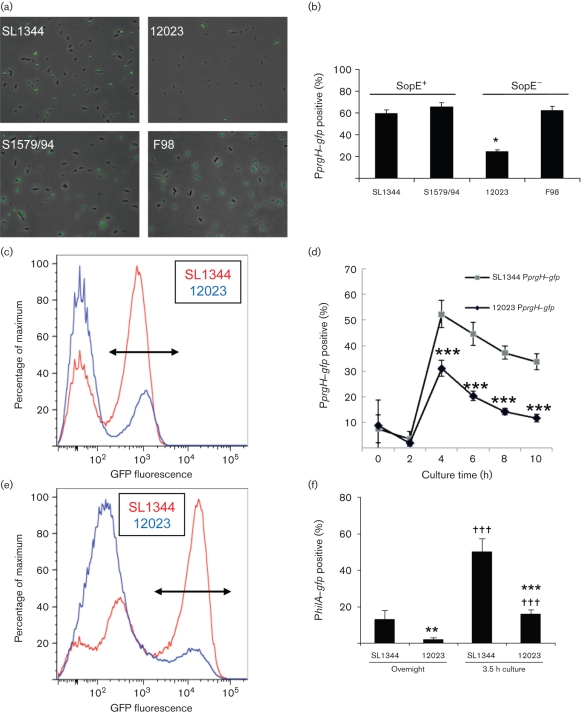
Comparison of P*prgH–gfp* expression in *Salmonella* strains. (a) The P*prgH–gfp* reporter was transduced into each *S*. Typhimurium strain and the levels of GFP expression within populations of bacteria grown to late-exponential phase (conditions of optimal SPI-1 expression) were compared by wide-field microscopy. Composite GFP and phase-contrast images are shown: field of view 90 µm×68 µm. Note that a smaller proportion of GFP-expressing bacteria are present in 12023 than the other three strains. (b) Quantification of the proportion of each strain with significant GFP expression in three replicate experiments by microscopical examination highlights the significantly lower expression of P*prgH–gfp* in 12023. Data are expressed as means±sem; significant difference from SopE^+^ strains, assessed by one-way ANOVA (*P*<0.05), is indicated by an asterisk. (c) Flow-cytometry analysis of SL1344 and 12023 carrying P*prgH–gfp* confirmed that both strains exhibited bistable expression characteristics, the intensity (but not size) of the positive peak being similar for both strains. The fluorescence range selected for further quantification of P*prgH–gfp* expression is indicated by the double-headed arrow. (d) Quantification of P*prgH–gfp* expression showed a peak at 4 h followed by a decline. Data are expressed as means±sem (*n* = 5–7). Significant differences between SL1344 and 12023 *PprgH–gfp* expression are indicated by asterisks (***, *P*<0.0001). (e) A chromosomal GFP reporter construct reveals lower *hilA* promoter activity in 12023 compared to SL1344. Note that the high GFP-expressing subpopulation was the only one that changed between stationary and late-exponential-phase growth, and this peak (indicated by the double-headed arrow) was subsequently used to compare expression between SL1344 and 12023 under stationary (overnight) and late-exponential-phase (3.5 h at 150 r.p.m.) cultures in three to six replicate experiments (f). Data in (f) are expressed as means±sd. Significant differences from SL1344: **, *P*<0.01; ***, *P*<0.0001. Significant differences from stationary phase: †††, *P*<0.0001.

Having established that our identification of a subpopulation of SL1344 expressing GFP driven by the *prgH* promoter reflects a true phenotypic difference in the invasive ability of these individuals, we transduced the P*prgH–gfp* fusion from SL1344 into the chromosome of other *S.* Typhimurium strains and compared the levels of GFP expression in these strains by microscopy. This analysis revealed that 12023 P*prgH–gfp* has a lower proportion of GFP-expressing individuals (Fig. 3a, b). The proportion expressing detectable levels of GFP in the late-exponential phase was only 25 % for 12023 P*prgH–gfp* compared to 60–65 % for the more invasive strains (SL1344, S1579/94 and F98) carrying the same transcriptional reporter. The fluorescence intensities of the expressing populations of SL1344 and 12023 were indistinguishable by flow cytometry (Fig. 3c) and both strains exhibited similar bistable expression characteristics, confirming that P*prgH* appears to be regulated in an ‘all-or-none’ fashion. Infection of MDCK cells with 12023 P*prgH–gfp* confirmed that the *prgH*-positive population identified by GFP expression represented a true phenotypic subpopulation, 99.0±1.8 % of cell-associated 12023 being GFP-positive. The difference between SL1344 and 12023 in P*prgH* activity was confirmed with the alternative transcriptional reporter, *lacZ.* Use of a P*prgH–lacZ* reporter plasmid indicated that *prgH* expression in 12023 was 65.6±5.6 % that of SL1344 (*n* = 3; *P*<0.001).

We next tested the possibility that the difference in P*prgH* activity between 12023 and SL1344 might be due to the two strains being in a different growth phase at 3.5 h following subculture. Growth rates were indistinguishable for both strains over a 9 h time-course (not shown) and analysis of the proportion of SL1344 and 12023 expressing P*prgH–gfp* at 2 h intervals over a 10 h time-course by flow cytometry revealed that *prgH* expression by both strains peaked at 4 h and subsequently declined (Fig. 3d). The proportion of 12023 expressing P*prgH–gfp* was significantly lower than that of SL1344 at all time points from 4 h onwards. The similar growth characteristics and indistinguishable kinetics of *prgH* expression in both strains confirms their divergent SPI-1 expression characteristics. Thus, the low invasiveness of 12023 appears to be correlated with a lower proportion of this strain expressing SPI-1.

**Fig. 4.**
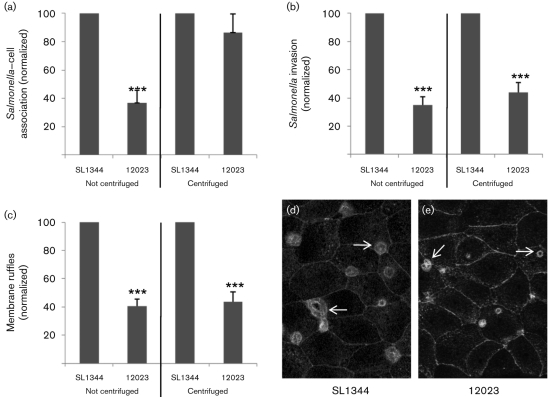
Centrifugation does not overcome the low invasiveness of 12023 compared to SL1344. *Salmonella* strains SL1344 and 12023 were grown to optimize invasiveness and used to infect MDCK cells grown on coverslips at an m.o.i. of ~20 for 15 min. Mild centrifugation (650 ***g***, 5 min, room temperature) was used to promote bacterial interaction with cells, and infection following this protocol was directly compared to our standard infection protocol, which does not include a centrifugation step. (a) All *Salmonella* associated with MDCK cells (adhered plus invaded) were enumerated by immunofluorescent labelling. Centrifugation overcomes the lower level of cell association exhibited by 12023 compared to SL1344. (b) *Salmonella* invasion measured by differential immunofluorescent labelling of adhered and invaded bacteria demonstrates that invasion by 12023 is unchanged and remains significantly lower than that of SL1344 following centrifugation. (c) Fluorescent labelling of membrane ruffles with TRITC-phalloidin reveals that the number of membrane ruffles generated by 12023 P*prgH–gfp* remains lower than that associated with SL1344 P*prgH–gfp* infection regardless of the use of centrifugation. In all graphs data are normalized to SL1344 and expressed as means±sem (*n* = 3–5). Significant differences from SL1344 are indicated by asterisks: ***, *P*<0.0001. (d, e) Confocal images of membrane ruffles induced during 15 min infection by SL1344 (d) or 12023 (e) following centrifugation. Note that 12023 induces fewer and smaller membrane ruffles. In both confocal images white arrows indicate the position of some *Salmonella*-induced membrane ruffles; field of view 110 µm×80 µm.

Motility of all strains was indistinguishable by microscopic analysis under the growth conditions under which P*prgH–gfp* analysis was performed. To discount the possibility that subtle motility differences or the expression of SPI-1-independent adhesins might contribute to the lower invasion of 12023 compared to SL1344 infection, experiments were repeated with a mild centrifugation to promote interaction of *Salmonella* with MDCK cells. The outcome of this infection protocol was directly compared to our standard protocol, which lacks a centrifugation step. Centrifugation promoted cell association and overcame the lower levels of bacterial interaction with MDCK cells seen with 12023 compared with SL1344 following our standard (non-centrifugation) protocol (Fig. 4a). Despite the numbers of strain 12023 and SL1344 associated with cells (adhered plus invaded) being comparable when centrifugation was used, invasion by 12023 was unchanged and remained significantly lower than that of SL1344 (Fig. 4b; *P*<0.0001). Enumeration of membrane ruffles induced by P*prgH–gfp* expressing SL1344 and 12023 as a secondary measure of SPI-1 activity confirmed that centrifugation had no effect on the behaviour of SL1344 and 12023, the numbers and size of membrane ruffles induced by 12023 remaining significantly lower (*P*<0.0001) than those induced by SL1344 (Fig. 4c–e). By discounting the possible contribution of additional adherence and motility factors, these data provide strong support for our conclusion that the observed difference in SPI-1 gene expression is the dominant factor underlying the lower invasiveness of 12023. Specificity of the SPI-1 expression difference was confirmed using a plasmid reporter expressing destabilized GFP under control of the *fliC* promoter, which revealed identical expression kinetics of *fliC* in SL1344 and 12023. Expression peaked 2 h after subculture, when 33.4±4.3 % (SL1344) and 34.3±3.9 % (12023) of the *Salmonella* populations were GFP-positive, and declined thereafter. The peak in P*fliC* expression preceded that of SPI-1 in both strains, confirming the recently described temporal hierarchy in flagellar and SPI-1 regulation in strain 14028/12023 (Saini *et al.*, 2010).

The striking difference in the proportion of individuals expressing GFP driven by the *prgH* promoter has important implications for researchers examining *Salmonella*–cell interactions at the single-cell level since it suggests that most of the 12023 population is unable to invade under standard conditions whereas most individuals of other *S.* Typhimurium strains tested are invasive. We confirmed our findings on 12023 using the equivalent strain from an alternative strain collection, ATCC 14028. Under identical assay conditions, 14028 exhibited similar levels of invasiveness to 12023 (0.44±0.11 ATCC 14028 per cell compared with 0.47±0.08 NCTC 12023 per cell). Transfer of the P*prgH–gfp* construct to 14028 revealed that a minority of this strain (22.8±3.2 %) expresses GFP, in agreement with results obtained with 12023 (24.6±2.7 %). Thus, our finding that low invasiveness correlates with low *prgH* expression in 12023 is validated by repetition in the equivalent strain acquired from an alternative source.

### Expression of a P*hilA–gfp* reporter correlates with *prgH* promoter activity in *S.* Typhimurium strains SL1344 and 12023

We next examined whether the low expression of *prgH* and invasiveness of 12023 might be related to decreased expression of the SPI-1-encoded transcriptional activator HilA. A P*hilA–gfp* chromosomal reporter was constructed based on a modified version of the P*prgH–gfp^+^* reporter (Hautefort *et al.*, 2003) in which P*prgH* was exchanged for P*hilA* and chloramphenicol resistance was exchanged for kanamycin resistance. This was incorporated into the chromosome of SL1344 using the Lambda Red method as previously performed with P*prgH–gfp*, since our experience with *prgH* reporters had demonstrated that chromosomal expression avoided additional effects of plasmid carriage on SPI-1 expression (Clark *et al.*, 2009).

Strains with the P*hilA–gfp* reporter integrated into the chromosome of SL1344 and 12023 exhibited heterogeneous expression (Fig. 3e), with profiles distinct from that of P*prgH* (Fig. 3c). Fluorescent peaks were less clearly defined than the P*prgH–gfp* profiles, with a substantial subpopulation expressing low levels of *PhilA–gfp* (Fig. 3e). A distinct subpopulation of varying size exhibited GFP expression levels at least an order of magnitude higher than the previously characterized P*prgH–gfp* positive population (Fig. 3e). Comparison between stationary (overnight) and late-exponential phase (3.5 h at 150 r.p.m.) cultures of both SL1344 and 12023 revealed that only the subpopulation with highest P*hilA–gfp* expression increased significantly during culture (data not shown). The discrete population with high P*hilA–gfp* expression was significantly larger in SL1344 than in 12023 in both stationary and late-exponential phases (Fig. 3e, f).

## DISCUSSION

Our data on *prgH* expression add to the growing body of evidence that only a subpopulation of *Salmonella* expresses SPI-1 genes, even under invasion-inducing conditions (Boddicker & Jones, 2004; Clark *et al.*, 2009; Hautefort *et al.*, 2003; Ibarra *et al.*, 2010). We have also confirmed that our measurement criteria for P*prgH–gfp* expression correspond with the ability of individual *Salmonella* to trigger membrane ruffles, a SPI-1 dependent phenotype. The existence of discrete subpopulations differing in their expression of SPI-1 is a clear example of bistable gene expression, which may allow *Salmonella* populations to adapt to and exploit changing conditions in the environment and host. Such marked heterogeneity in gene expression has important implications for studies that examine infection at the single-cell level since the behaviour of individual bacteria will differ markedly. The ability of membrane ruffles to internalize the SPI-1-non-expressing organisms within the population has additional implications for single-cell analyses since SPI-1 promotes intracellular proliferation (Steele-Mortimer *et al.*, 2002) and SPI-1 effectors such as SigD/SopB control intracellular trafficking and accelerate replication (Bujny *et al.*, 2008; Hernandez *et al.*, 2004). It should also be noted that other studies have suggested that the downregulation of SPI-1 genes after uptake enhances survival and replication (Boddicker & Jones, 2004). Thus, *Salmonella* populations might be expected to behave heterogeneously during infection of host cells due to differential expression of SPI-1 and its effectors.

Comparing P*prgH–gfp^+^* expression in different strains revealed a previously unsuspected level of heterogeneity, since SL1344 and two other *S*. Typhimurium strains (S1579/97 and F98) mostly had *prgH* expression ‘switched on’ during late-exponential-phase growth, whilst a minority of the other commonly studied *S.* Typhimurium strain 12023/14028 expressed this gene under identical conditions, in line with its reduced invasiveness. The potential impact of strain heterogeneity should be considered when comparing studies with different strains as, clearly, *S.* Typhimurium strains differ with respect to SPI-1 regulation and invasion. The close correlation between P*prgH* expression levels and epithelial infection that we observed both at population level and by single-cell analysis suggested that the lower expression of SPI-1 genes in 12023 is the major factor in its reduced invasion. Although these data cannot exclude the possible involvement of additional factors in the distinct invasiveness of 12023, our data on motility, *fliC* expression and the effects of centrifugation allowed us to discount differences in motility or other factors involved in the initial interaction of *Salmonella* with epithelial cells as potential contributory factors. Furthermore, since bacterial adherence was closely correlated with P*prgH* activity, and membrane ruffle induction mirrored the relative invasion levels of the two strains, the evidence is compelling that the observed difference in SPI-1 expression is at least the primary factor underlying the different levels of invasion. The finding that expression of a P*hilA–gfp* reporter mirrors that of P*prgH–gfp* in 12023 and SL1344 implicates differences in operation of one or more regulatory mechanisms acting via the HilA transcription factor in the prominent difference in invasiveness between these strains. It is also noteworthy that SL1344 and 12023 exhibited similar upregulation of *prgH* and *hilA* 3–4 h following subculture, indicating that the growth-phase-dependent regulation operates in a largely similar fashion in both strains. This conclusion was further supported by the similar growth characteristics of each strain and the fact that activity of the growth-phase-dependent *fliC* promoter within subpopulations of SL1344 and 12023 was indistinguishable. Studies aiming to dissect the SPI-1 regulatory networks should now consider the existence of strains naturally differing in SPI-1 regulation to refine regulatory models and explain the distinctive behaviour of individual strains.

We have demonstrated that absence of SopE is consistently associated with decreases in both the speed at which membrane ruffles are induced and their size in all strains examined, extending previous data on the effects of SopE deletion from SL1344 in infection of other cell lines (Hardt *et al.*, 1998; Stender *et al.*, 2000). Somewhat surprisingly, the clear association between SopE status and membrane ruffling was not mirrored by a consistent effect of SopE on *Salmonella* invasiveness, although we did confirm data from distinct cell lines that deletion of *sopE* from SL1344 reduces invasiveness (Hardt *et al.*, 1998; Mirold *et al.*, 2001; Stender *et al.*, 2000; Zhou *et al.*, 2001). The heterogeneity in impact of SopE is highlighted by the fact that 12023 and SL1344 *sopE*^−^ induced ruffles of similarly reduced size and kinetics as other SopE^−^ strains but were the only SopE^−^ strains that exhibited lower invasion, the defect in invasion being much more prominent in the case of 12023 due to its lower expression of SPI-1. Together our data demonstrate that careful examination of ruffling dynamics and morphology can reveal effector roles that are more subtle than those associated with prominent invasion defects. Our finding that there is heterogeneity between strains with respect to the role of SopE reiterates our previous findings that the role of another SPI-1 effector, SipA, on membrane ruffling varies between different wild-type strains (Perrett & Jepson, 2009) and highlights the difficulty in extrapolating data on effector function between *Salmonella* strains.

The finding that the role of SopE in membrane ruffling does not always affect invasion suggests that the processes of membrane ruffling and invasion can to some extent be uncoupled. This observation might at first appear counterintuitive, the ability of *Salmonella*-induced membrane ruffles to internalize both invasive and non-invasive bacteria (Francis *et al.*, 1993) having been widely interpreted as suggesting that ruffling and invasion are inseparable. However, evidence from a number of studies suggests that this causal relationship is less straightforward. For example, deletion of *sipA* decreases invasion without overtly affecting ruffling, suggesting that SipA has a role in invasion that is to some extent separate from membrane ruffling (Jepson *et al.*, 2001). Furthermore, Hänisch *et al.* (2010) recently reported that inhibition of membrane ruffling by interference with WAVE complex function does not always lead to a significant decrease in *Salmonella* invasion. Thus, while membrane ruffling probably has a major role in promoting invasion, especially when there is sufficient contact of additional bacteria with membrane ruffles for passive ‘secondary’ uptake to make a significant contribution to total uptake of bacteria, additional entry mechanisms operate that are separate from the macropinocytic activity associated with ruffling. These findings indicate that there are additional levels of complexity in the invasion process that have until recently been overlooked.

## References

[r1] Boddicker J. D. Jones B. D. **(**2004**).** Lon protease activity causes down-regulation of *Salmonella* pathogenicity island 1 invasion gene expression after infection of epithelial cells. Infect Immun 72, 2002–2013. 10.1128/IAI.72.4.2002-2013.2004.. 15039320 PMC375200

[r2] Bruno V. M. Hannemann S. Lara-Tejero M. Flavell R. A. Kleinstein S. H. Galán J. E. **(**2009**).** *Salmonella* Typhimurium type III secretion effectors stimulate innate immune responses in cultured epithelial cells. PLoS Pathog 5, e1000538. 10.1371/journal.ppat.1000538.. 19662166 PMC2714975

[r3] Bujny M. V. Ewels P. A. Humphrey S. Attar N. Jepson M. A. Cullen P. J. **(**2008**).** Sorting nexin-1 defines an early phase of *Salmonella*-containing vacuole-remodeling during *Salmonella* infection. J Cell Sci 121, 2027–2036. 10.1242/jcs.018432.. 18505799

[r4] Clark L. Martinez-Argudo I. Humphrey T. J. Jepson M. A. **(**2009**).** GFP plasmid-induced defects in *Salmonella* invasion depend on plasmid architecture, not protein expression. Microbiology 155, 461–467. 10.1099/mic.0.025700-0.. 19202094

[r5] Datsenko K. A. Wanner B. L. **(**2000**).** One-step inactivation of chromosomal genes in *Escherichia coli* K-12 using PCR products. Proc Natl Acad Sci U S A 97, 6640–6645. 10.1073/pnas.120163297.. 10829079 PMC18686

[r6] Ellermeier J. R. Slauch J. M. **(**2007**).** Adaptation to the host environment: regulation of the SPI1 type III secretion system in *Salmonella enterica* serovar Typhimurium. Curr Opin Microbiol 10, 24–29. 10.1016/j.mib.2006.12.002.. 17208038

[r7] Francis C. L. Ryan T. A. Jones B. D. Smith S. J. Falkow S. **(**1993**).** Ruffles induced by *Salmonella* and other stimuli direct macropinocytosis of bacteria. Nature 364, 639–642. 10.1038/364639a0.. 8350922

[r8] Fu Y. Galán J. E. **(**1999**).** A *Salmonella* protein antagonizes Rac-1 and Cdc42 to mediate host-cell recovery after bacterial invasion. Nature 401, 293–297. 10.1038/45829.. 10499590

[r9] Hänisch J. Ehinger J. Ladwein M. Rohde M. Derivery E. Bosse T. Steffen A. Bumann D. Misselwitz B. **(**2010**).** Molecular dissection of *Salmonella*-induced membrane ruffling versus invasion. Cell Microbiol 12, 84–98. 10.1111/j.1462-5822.2009.01380.x.. 19732055

[r10] Hardt W. D. Chen L. M. Schuebel K. E. Bustelo X. R. Galán J. E. **(**1998**).** *S. typhimurium* encodes an activator of Rho GTPases that induces membrane ruffling and nuclear responses in host cells. Cell 93, 815–826. 10.1016/S0092-8674(00)81442-7.. 9630225

[r11] Hautefort I. Proença M. J. Hinton J. C. **(**2003**).** Single-copy green fluorescent protein gene fusions allow accurate measurement of *Salmonella* gene expression in vitro and during infection of mammalian cells. Appl Environ Microbiol 69, 7480–7491. 10.1128/AEM.69.12.7480-7491.2003.. 14660401 PMC310007

[r12] Hayward R. D. Koronakis V. **(**1999**).** Direct nucleation and bundling of actin by the SipC protein of invasive *Salmonella*. EMBO J 18, 4926–4934. 10.1093/emboj/18.18.4926.. 10487745 PMC1171564

[r13] Henry T. García-Del Portillo F. Gorvel J. P. **(**2005**).** Identification of *Salmonella* functions critical for bacterial cell division within eukaryotic cells. Mol Microbiol 56, 252–267. 10.1111/j.1365-2958.2005.04540.x.. 15773994

[r14] Hernandez L. D. Hueffer K. Wenk M. R. Galán J. E. **(**2004**).** *Salmonella* modulates vesicular traffic by altering phosphoinositide metabolism. Science 304, 1805–1807. 10.1126/science.1098188.. 15205533

[r15] Hoiseth S. K. Stocker B. A. **(**1981**).** Aromatic-dependent *Salmonella typhimurium* are non-virulent and effective as live vaccines. Nature 291, 238–239. 10.1038/291238a0.. 7015147

[r16] Humphrey S. Clark L. F. Humphrey T. J. Jepson M. A. **(**2011**).** Enhanced recovery of *Salmonella* Typhimurium DT104 from exposure to stress at low temperature. Microbiology 157, 1103–1114.. 21178168 10.1099/mic.0.045666-0

[r17] Ibarra J. A. Knodler L. A. Sturdevant D. E. Virtaneva K. Carmody A. B. Fischer E. R. Porcella S. F. Steele-Mortimer O. **(**2010**).** Induction of *Salmonella* pathogenicity island 1 under different growth conditions can affect *Salmonella*-host cell interactions *in vitro*. Microbiology 156, 1120–1133. 10.1099/mic.0.032896-0.. 20035008 PMC2848694

[r18] Jepson M. A. Kenny B. Leard A. D. **(**2001**).** Role of *sipA* in the early stages of *Salmonella typhimurium* entry into epithelial cells. Cell Microbiol 3, 417–426. 10.1046/j.1462-5822.2001.00124.x.. 11422084

[r19] Klein J. R. Fahlen T. F. Jones B. D. **(**2000**).** Transcriptional organization and function of invasion genes within *Salmonella enterica* serovar Typhimurium pathogenicity island 1, including the *prgH*, *prgI*, *prgJ*, *prgK*, *orgA*, *orgB*, and *orgC* genes. Infect Immun 68, 3368–3376. 10.1128/IAI.68.6.3368-3376.2000.. 10816487 PMC97603

[r20] Kubori T. Matsushima Y. Nakamura D. Uralil J. Lara-Tejero M. Sukhan A. Galán J. E. Aizawa S. I. **(**1998**).** Supramolecular structure of the *Salmonella typhimurium* type III protein secretion system. Science 280, 602–605. 10.1126/science.280.5363.602.. 9554854

[r21] Lara-Tejero M. Galán J. E. **(**2009**).** *Salmonella enterica* serovar Typhimurium pathogenicity island 1-encoded type III secretion system translocases mediate intimate attachment to nonphagocytic cells. Infect Immun 77, 2635–2642. 10.1128/IAI.00077-09.. 19364837 PMC2708559

[r22] Linn T. St Pierre R. **(**1990**).** Improved vector system for constructing transcriptional fusions that ensures independent translation of *lacZ*. J Bacteriol 172, 1077–1084.. 2137119 10.1128/jb.172.2.1077-1084.1990 PMC208539

[r23] McGhie E. J. Hayward R. D. Koronakis V. **(**2001**).** Cooperation between actin-binding proteins of invasive *Salmonella*: SipA potentiates SipC nucleation and bundling of actin. EMBO J 20, 2131–2139. 10.1093/emboj/20.9.2131.. 11331579 PMC125241

[r24] McGhie E. J. Hayward R. D. Koronakis V. **(**2004**).** Control of actin turnover by a *Salmonella* invasion protein. Mol Cell 13, 497–510. 10.1016/S1097-2765(04)00053-X.. 14992720

[r25] McGhie E. J. Brawn L. C. Hume P. J. Humphreys D. Koronakis V. **(**2009**).** *Salmonella* takes control: effector-driven manipulation of the host. Curr Opin Microbiol 12, 117–124. 10.1016/j.mib.2008.12.001.. 19157959 PMC2647982

[r26] Miller J. H. **(**1972**).** Experiments in Molecular Genetics. Cold Spring Harbor, NY: Cold Spring Harbor Laboratory.

[r27] Mirold S. Ehrbar K. Weissmüller A. Prager R. Tschäpe H. Rüssmann H. Hardt W. D. **(**2001**).** *Salmonella* host cell invasion emerged by acquisition of a mosaic of separate genetic elements, including *Salmonella* pathogenicity island 1 (SPI1), SPI5, and *sopE2*. J Bacteriol 183, 2348–2358. 10.1128/JB.183.7.2348-2358.2001.. 11244077 PMC95144

[r28] Misselwitz B. Kreibich S. K. Rout S. Stecher B. Periaswamy B. Hardt W. D. **(**2011**).** *Salmonella enterica* serovar Typhimurium binds to HeLa cells via Fim-mediated reversible adhesion and irreversible type three secretion system 1-mediated docking. Infect Immun 79, 330–341. 10.1128/IAI.00581-10.. 20974826 PMC3019867

[r29] Müller A. J. Hoffmann C. Galle M. Van Den Broeke A. Heikenwalder M. Falter L. Misselwitz B. Kremer M. Beyaert R. Hardt W. D. **(**2009**).** The *S.* Typhimurium effector SopE induces caspase-1 activation in stromal cells to initiate gut inflammation. Cell Host Microbe 6, 125–136. 10.1016/j.chom.2009.07.007.. 19683679

[r30] Norris F. A. Wilson M. P. Wallis T. S. Galyov E. E. Majerus P. W. **(**1998**).** SopB, a protein required for virulence of *Salmonella dublin*, is an inositol phosphate phosphatase. Proc Natl Acad Sci U S A 95, 14057–14059. 10.1073/pnas.95.24.14057.. 9826652 PMC24325

[r31] Patel J. C. Galán J. E. **(**2006**).** Differential activation and function of Rho GTPases during *Salmonella*-host cell interactions. J Cell Biol 175, 453–463. 10.1083/jcb.200605144.. 17074883 PMC2064522

[r32] Perrett C. A. Jepson M. A. **(**2007**).** Applications of cell imaging in *Salmonella* research. Methods Mol Biol 394, 235–273. 10.1007/978-1-59745-512-1_12.. 18363239

[r33] Perrett C. A. Jepson M. A. **(**2009**).** Regulation of *Salmonella*-induced membrane ruffling by SipA differs in strains lacking other effectors. Cell Microbiol 11, 475–487. 10.1111/j.1462-5822.2008.01268.x.. 19046340

[r34] Perrett C. A. Karavolos M. H. Humphrey S. Mastroeni P. Martinez-Argudo I. Spencer H. Bulmer D. Winzer K. McGhie E. **(**2009**).** LuxS-based quorum sensing does not affect the ability of *Salmonella enterica* serovar Typhimurium to express the SPI-1 type 3 secretion system, induce membrane ruffles, or invade epithelial cells. J Bacteriol 191, 7253–7259. 10.1128/JB.00727-09.. 19783624 PMC2786567

[r35] Raffatellu M. Wilson R. P. Chessa D. Andrews-Polymenis H. Tran Q. T. Lawhon S. Khare S. Adams L. G. Bäumler A. J. **(**2005**).** SipA, SopA, SopB, SopD, and SopE2 contribute to *Salmonella enterica* serotype Typhimurium invasion of epithelial cells. Infect Immun 73, 146–154. 10.1128/IAI.73.1.146-154.2005.. 15618149 PMC538951

[r36] Saini S. Slauch J. M. Aldridge P. D. Rao C. V. **(**2010**).** Role of cross talk in regulating the dynamic expression of the flagellar *Salmonella* pathogenicity island 1 and type 1 fimbrial genes. J Bacteriol 192, 5767–5777. 10.1128/JB.00624-10.. 20833811 PMC2953706

[r37] Schraidt O. Lefebre M. D. Brunner M. J. Schmied W. H. Schmidt A. Radics J. Mechtler K. Galán J. E. Marlovits T. C. **(**2010**).** Topology and organization of the *Salmonella typhimurium* type III secretion needle complex components. PLoS Pathog 6, e1000824. 10.1371/journal.ppat.1000824.. 20368966 PMC2848554

[r38] Steele-Mortimer O. Brumell J. H. Knodler L. A. Méresse S. Lopez A. Finlay B. B. **(**2002**).** The invasion-associated type III secretion system of *Salmonella enterica* serovar Typhimurium is necessary for intracellular proliferation and vacuole biogenesis in epithelial cells. Cell Microbiol 4, 43–54. 10.1046/j.1462-5822.2002.00170.x.. 11856172

[r39] Stender S. Friebel A. Linder S. Rohde M. Mirold S. Hardt W. D. **(**2000**).** Identification of SopE2 from *Salmonella typhimurium*, a conserved guanine nucleotide exchange factor for Cdc42 of the host cell. Mol Microbiol 36, 1206–1221. 10.1046/j.1365-2958.2000.01933.x.. 10931274

[r40] Temme K. Salis H. Tullman-Ercek D. Levskaya A. Hong S. H. Voigt C. A. **(**2008**).** Induction and relaxation dynamics of the regulatory network controlling the type III secretion system encoded within *Salmonella* pathogenicity island 1. J Mol Biol 377, 47–61. 10.1016/j.jmb.2007.12.044.. 18242639 PMC2280070

[r41] Terebiznik M. R. Vieira O. V. Marcus S. L. Slade A. Yip C. M. Trimble W. S. Meyer T. Finlay B. B. Grinstein S. **(**2002**).** Elimination of host cell PtdIns(4,5)P(2) by bacterial SigD promotes membrane fission during invasion by *Salmonella*. Nat Cell Biol 4, 766–773. 10.1038/ncb854.. 12360287

[r42] Unsworth K. E. Way M. McNiven M. Machesky L. Holden D. W. **(**2004**).** Analysis of the mechanisms of *Salmonella*-induced actin assembly during invasion of host cells and intracellular replication. Cell Microbiol 6, 1041–1055. 10.1111/j.1462-5822.2004.00417.x.. 15469433

[r43] Zhang S. Santos R. L. Tsolis R. M. Mirold S. Hardt W. D. Adams L. G. Bäumler A. J. **(**2002**).** Phage mediated horizontal transfer of the *sopE1* gene increases enteropathogenicity of *Salmonella enterica* serotype Typhimurium for calves. FEMS Microbiol Lett 217, 243–247. 10.1111/j.1574-6968.2002.tb11482.x.. 12480111

[r44] Zhou D. Galán J. E. **(**2001**).** *Salmonella* entry into host cells: the work in concert of type III secreted effector proteins. Microbes Infect 3, 1293–1298. 10.1016/S1286-4579(01)01489-7.. 11755417

[r45] Zhou D. Mooseker M. S. Galán J. E. **(**1999**).** Role of the *S. typhimurium* actin-binding protein SipA in bacterial internalization. Science 283, 2092–2095. 10.1126/science.283.5410.2092.. 10092234

[r46] Zhou D. Chen L. M. Hernandez L. Shears S. B. Galán J. E. **(**2001**).** A *Salmonella* inositol polyphosphatase acts in conjunction with other bacterial effectors to promote host cell actin cytoskeleton rearrangements and bacterial internalization. Mol Microbiol 39, 248–259. 10.1046/j.1365-2958.2001.02230.x.. 11136447

